# The Impact of Socioeconomic Inequities and Small-Area Deprivation on Child Inpatient Care: Evidence from a Quantitative Study in a Vulnerable Suburban Setting

**DOI:** 10.3390/ijerph23060767

**Published:** 2026-06-07

**Authors:** Tânia Russo, João Pereira

**Affiliations:** 1 NOVA National School of Public Health (ENSP), NOVA University Lisbon, Avenida Padre Cruz, 1600-560 Lisbon, Portugal; jpereira@ensp.unl.pt; 2 Department of Pediatrics, Hospital Prof. Doutor Fernando Fonseca, Unidade Local de Saúde Amadora/Sintra, IC 19, 2720-276 Amadora, Portugal; 3 Public Health Research Center (CISP), Comprehensive Health Research Center (CHRC), REAL, CCAL, NOVA National School of Public Health (ENSP), NOVA University Lisbon, Avenida Padre Cruz, 1600-560 Lisbon, Portugal

**Keywords:** health inequities, social determinants of health, ethnicity, pediatric intensive care, hospitalization, children, Portugal

## Abstract

**Highlights:**

**Public health relevance—How does this work relate to a public health issue?**

- Socioeconomic determinants of child health have long been recognized but their importance is often overlooked.

**Public health significance—Why is this work of significance to public health?**

- Inequities in pediatric hospital stay and ICU care found in small-level deprived areas of Lisbon suburban municipalities.
- Ethnicity, low maternal education, and low-skilled parents are linked to higher hospital care needs.

**Public health implications—What are the key implications or messages for practitioners, policy makers and/or researchers in public health?**

- Socioeconomic inequities are associated with longer hospital stay and clinical severity in children.
- Early-life public health and social policies are key to breaking adversity cycles.

**Abstract:**

Socioeconomic inequities are associated with longer length of hospital stay (LOS) and clinical severity in children. This retrospective cross-sectional study analyzes health inequalities and area-level deprivation, focusing on hospitalization indicators, in a suburban pediatric population in Portugal. Pediatric admissions to a local general hospital were analyzed for LOS and admission to intensive care unit (ICU), as well as their relation to socioeconomic factors, over an 8-year period (2014 to 2021), using population-averaged models. Area-level inequalities were measured for the population ranked by civil parishes’ European Deprivation Index. 8016 admissions were included. Health inequalities associated with socioeconomic deprivation were observed, with concentration curves above the diagonal for LOS and admission to ICU and located in urban and densely populated civil parishes. Neonatal age showed the highest mean LOS ratio (MR = 2.29, 95% CI 1.96; 2.67, *p* < 0.001) and ICU admission odds (OR = 9.25, 95% CI 4.84; 17.68, *p* < 0.001). Mean LOS ratio was significantly higher for Black ethnicity (MR = 1.19; 95% CI 1.10; 1.28, *p* < 0.001) and lower maternal education. Odds of admission to ICU was significantly higher for male gender (OR = 1.25, 95% CI 1.01; 1.55, *p* = 0.048) and mother’s unskilled occupation (OR = 1.66, 95% CI 1.09; 2.53, *p* = 0.019). Paternal manual skilled occupation demonstrated 17% higher mean LOS ratio (*p* < 0.001) and 51% higher odds of admission to ICU (*p* = 0.019). Public policies must be culturally competent and target socioeconomic and geographical deprivation.

## 1. Introduction

Health and health care are strongly influenced by the social and economic conditions in which individuals are born, grow, live, work, and age [1]. These conditions are unevenly distributed within and across communities and countries, contributing to substantial differences in health outcomes and access to care. Across diverse settings worldwide, a persistent social gradient in health has been documented, whereby individuals with lower socioeconomic status experience poorer health outcomes, higher disease burden, and greater health care needs [1]. These systematic differences between population groups are referred to as health inequities and are widely recognized as avoidable and socially unjust [2].

Over recent decades, increasing scientific evidence and policy attention have highlighted the importance of addressing the social determinants of health to reduce these inequities. Publications such as the report of the Commission on Social Determinants of Health by the World Health Organization have contributed to placing health inequities at the center of the global public health agenda [1]. Despite progress in reducing poverty and socioeconomic inequality over the past two decades, inequities remain a significant challenge in Portugal. Within the European Union (EU), Portugal continues to rank among the most unequal countries, with a Gini coefficient of 0.319 in 2023, placing it as the fifth most unequal Member State [3].

Socioeconomic disparities exert their influence across the life course, beginning before conception and extending through adulthood and across generations [4]. Children are particularly vulnerable to the socioeconomic conditions of their caregivers, including income, education, occupation, lifestyle, housing conditions, and neighborhood environments. These factors shape early-life exposures and opportunities, which may have lasting effects on health and development.

Health inequalities associated with socioeconomic deprivation have been documented in several hospital care indicators, including hospitalization rates, intensive care utilization, and length of hospital stay (LOS) [5,6,7,8,9]. Inpatient care indicators such as LOS and admission to intensive care units (ICU) reflect the clinical severity and health needs of patients as assessed by physicians. Consequently, systematic differences in these indicators according to socioeconomic conditions may reflect underlying inequities in health needs [10].

The municipalities of Amadora and Sintra, located on the outskirts of Lisbon, are characterized by considerable socioeconomic vulnerability [11,12]. Together, they comprised 557,060 residents in 2021 and have relatively young populations, with 26.2% aged between 0 and 24 years [13]. Immigration has also contributed to marked ethnic and cultural diversity in these municipalities, where 10.3% of residents were of foreign origin in 2021 [14]. Reflecting Portugal’s colonial history, the most frequent country of origin is Brazil (32.9%), and 42.6% of immigrants originate from Portuguese-speaking African countries [14]. As a result, ethnic minority groups are more prevalent in these municipalities than in the country overall [14,15].

Local health care provision includes one general public hospital, 10 primary health care units in Amadora, and 28 in Sintra. However, access to primary health care remains substantially constrained in these municipalities, with 29.4% of residents registered in primary health care centers not having been assigned a family doctor [16]. As a result, many residents rely heavily on hospital emergency services for health care access.

Research examining health inequities in the pediatric population in Portugal remains scarce and has largely focused on specific subpopulations [17,18,19]. Although some studies have explored the impact of social determinants of health on immigrant children and children of African descent living in Amadora [20,21,22,23,24,25], the municipality of Sintra—despite being more populous and sharing similar socioeconomic characteristics and health care resources—has rarely been examined in this context.

Therefore, the present study aims to analyze health inequalities among children residing in Amadora and Sintra who were hospitalized in the local public hospital between 2014 and 2021, and to investigate the association between hospital care needs and area-level socioeconomic deprivation.

## 2. Materials and Methods

### 2.1. Study Design and Population

This retrospective cross-sectional study analyzed hospital admissions to the Department of Pediatrics of the local general public hospital among patients residing in the municipalities of Amadora and Sintra, Portugal, between 1 January 2014 and 31 December 2021. The unit of observation was an individual hospital admission.

Admissions to the pediatric ward, short-term observation unit, neonatal ICU and pediatric ICU were included. The following exclusion criteria were applied: (i) residence outside the municipalities of Amadora or Sintra; (ii) missing information on civil parish of residence; (iii) missing or unknown information regarding both parents; and (iv) missing data for all socioeconomic variables examined.

Data were obtained from hospital administrative and clinical records. Socioeconomic information was reported by caregivers and collected by nursing staff through direct interview at the time of admission.

### 2.2. Dependent Variables: Hospitalization Indicators

Two dependent variables were used as indicators of needed hospital care:

- Length of stay (LOS), measured as a continuous variable representing the number of days of hospitalization.
- Admission to an intensive care unit (ICU), defined as a binary variable.

The first ward of admission was considered for analysis, even when patients were subsequently transferred to another ward during hospitalization.

### 2.3. Independent Variables: Demographic and Socioeconomic Factors

Demographic and socioeconomic variables were selected according to the PROGRESS framework [26]. The following variables were included: civil parish of residence; age group; gender; ethnicity; main language spoken in the household; mother’s and father’s education level; mother’s and father’s occupation.

Age was categorized into ordinal age groups according to international definitions [27]. Admissions of patients older than 18 years were included when these individuals were followed in the Department of Pediatrics for chronic conditions.

Ethnicity categories for Black and Asian populations included children of mixed Black-White and mixed Asian backgrounds, respectively.

Parental education level was classified according to the Portuguese Basic Law of the Educational System into: first cycle of basic education (4 years); second cycle of basic education (2 years); third cycle of basic education (3 years); secondary education (3 years); higher education (bachelor’s, master’s, or doctoral degree) [28]. Compulsory schooling in Portugal was extended to secondary education or 18 years of age in 2009 [29].

Parental occupations were classified according to the Portuguese Classification of Occupations, which includes 10 occupational groups [30]. These groups were further aggregated into four broader categories: white-collar workers; armed forces, security, personal care, and sales workers; skilled manual workers; unskilled workers. The not-working category included unemployed individuals, students not engaged in employment, and prison detainees. Individuals simultaneously working and studying were classified as working.

### 2.4. Missing Data

Table S1 (Supplementary Materials) presents the proportion of missing cases for each independent socioeconomic variable. After excluding cases with missing data for all socioeconomic variables, remaining missing values were handled using multiple imputation, assuming that data were missing at random (MAR).

Imputation was performed using fully conditional specification, with a maximum of 10 iterations per imputation cycle [31,32]. Logistic regression models were applied for categorical variables, whereas predictive mean matching (PMM) was used for continuous variables. PMM selected the 10 closest observed cases without missing values. This method is less sensitive than linear regression to non-normal distribution [32].

All variables included in the analysis models were also included in the imputation model: age, gender, LOS, ICU admission, language, parental education level, and parental occupation.

The number of imputations performed was set to at least equal the percentage of incomplete cases [32]; therefore, 40 imputations were generated. Multiple imputation procedures were conducted using IBM^®^ SPSS^®^ Statistics version 30.0.0.0.

### 2.5. Data Analysis

Descriptive analyses included boxplots and histograms to assess the distribution of the continuous dependent variable (LOS). Normality was tested using the Kolmogorov–Smirnov test.

#### 2.5.1. Multivariate Analysis

Explanatory models were built. To account for correlation arising from repeated admissions by individuals, generalized estimating equations (GEE) with repeated measures were used (patient identifier as clustering unit (“subject”); exchangeable working structure; robust estimation). As a non-normal distribution was shown, LOS was modelled using a gamma distribution with log link function, for a positively skewed distribution. ICU admission was modelled with a binomial distribution and logit link function. These models allow estimation of mean ratios and odds ratios, respectively. Exponentiated coefficients (Exp(β)) are therefore reported as mean ratios (MR) for LOS and as odds ratios (OR) for ICU admission, each with 95% confidence intervals (CIs). The models included all the demographic and socioeconomic variables studied, with year of admission included to adjust for temporal variation in clinical practice and case mix over the study period (fixed year effects).

Statistical significance was defined as *p* < 0.05. All analyses were conducted using IBM^®^ SPSS^®^ Statistics version 30.0.0.0.

#### 2.5.2. Health Equity Analysis

Health inequity was defined as the systematic association between needed hospital care and socioeconomic deprivation. Investigation of whether such inequity exists, and to what extent, was assessed through ecological small-area analysis using the civil parish of residence of hospitalized children.

Socioeconomic inequality in LOS and ICU admission was evaluated using concentration curves and indices [33,34]. Concentration curves were constructed by plotting the cumulative proportion of the population aged 0–24 years ranked by deprivation level against the cumulative proportion of LOS or ICU admissions [10,35].

Socioeconomic deprivation was measured using the Portuguese version of the European Deprivation Index (EDI-PT), derived from the national 2011 Census and calculated for each civil parish (Table S2 in Supplementary Materials). Scores were updated according to the administrative reorganization of civil parishes implemented in 2013 [36,37].

Concentration curves and indices were calculated using Microsoft Excel^®^ (Microsoft 365^®^, version 2403). Concentration indices were estimated using a linear approximation method based on grouped data [10].

#### 2.5.3. Geographical Distribution of Deprivation and Hospital Care Needs

EDI-PT scores for the 17 civil parishes comprising the municipalities of Amadora and Sintra were ranked and divided into quintiles. These quintiles were based on the distribution of EDI-PT scores within the study area and therefore differ from national-level quintiles.

The population-based rates of LOS and ICU admissions for each civil parish were calculated as the ratio between the respective totals and the number of residents aged 0–24 years in the corresponding civil parish. These rates were subsequently ranked and categorized into quintiles.

The small-area distribution of these two indicators, together with the deprivation index, was mapped at the civil parish level using quintile categories to assess the geographical correspondence between socioeconomic deprivation and hospital care need.

## 3. Results

**Figure 1:**
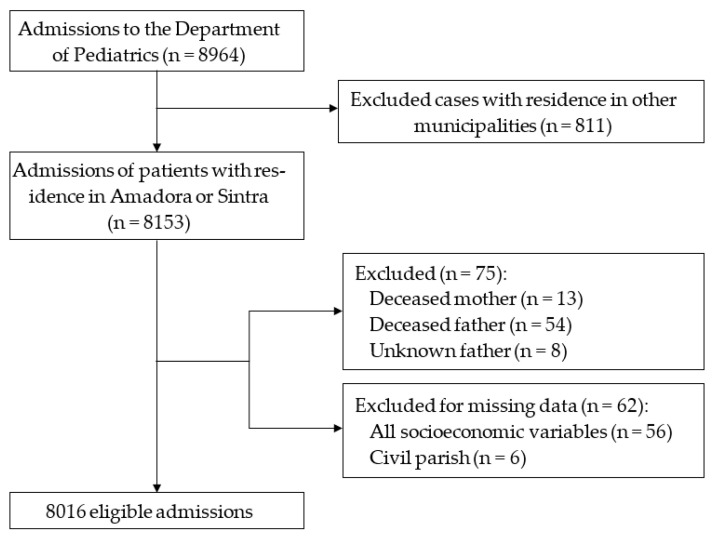
Process of case selection.

Between 2014 and 2021, a total of 8153 admissions to the hospital’s Department of Pediatrics involved patients residing in the municipalities of Amadora and Sintra. After applying the exclusion criteria, 137 cases were removed, resulting in a final analytical sample of 8016 hospital admissions (Figure 1), corresponding to 6088 individual patients.

During the study period, 1024 individuals (12.8%) experienced more than one hospital admission. The maximum number of admissions per individual was 27. The mean number of admissions per individual was 1.32 ± 1.14, with a median of 1.00 (interquartile range [IQR] 1.00–1.00). During the study period, 13 deaths were recorded.

Table 1 summarizes the demographic and socioeconomic characteristics of admissions. Fifty four percent of admissions were of male subjects. Age did not follow a normal distribution and ranged from 0.00 to 22.00 years, with mean 5.08 ± 5.74 years and median 2.30 years (IQR 0.40; 8.82). More than one third of the admissions were of very young children (less than 1 year old) and neonates represented 10.9% of the sample. Children were younger than 2 years in 47.4% of admissions. In only four admissions patients were 20 years or older (one was admitted to ICU).

Together, White and Black ethnicities comprised 96.8% of the sample. Portuguese was the main language spoken in the household, whilst African languages (Creole and Fulah) comprised 42.5% of the foreign languages reported. Both mothers and fathers had none or only basic education level in more than half of cases (mothers 52.5%, fathers 56.3%), with a slightly higher proportion of mothers (47.6% versus 43.7% of fathers) having secondary or higher level. Non-working status was very high among mothers (30.2%).

Residence in the municipality of Sintra represented 63.3% (n = 5076) of admissions. Residence in the most deprived civil parishes (fifth quintile EDI-PT score) accounted for 95.6% (n = 7663) of admissions.

The most frequent diagnostic groups were respiratory diseases (n = 2699, 33.7%) and acute gastroenteritis (n = 711, 8.9%), followed by neonatal conditions (n = 546, 6.8%) and sickle cell disease (n = 502, 6.3%). Together, these four diagnostic groups accounted for 55.6% of all admissions.

### 3.1. Length of Hospital Stay

LOS ranged from 0.65 to 246.00 days, with a mean of 6.47 ± 11.02 days and a median of 4.00 days (IQR 2.00; 7.00). Neither the variable LOS nor its log-transformation were normally distributed for any of the categories of the independent variables, as assessed by Kolmogorov-Smirnov test (*p* < 0.001).

In the GEE model for LOS (Table 2), adjusted for year of admission, age showed the strongest association. Compared with adolescents aged ≥ 15 years, neonatal admissions were associated with a significantly longer LOS, with a more than two-fold higher mean LOS (MR = 2.29, 95% CI 1.96; 2.67, *p* < 0.001). In contrast, children aged 1 to 4 years and 5 to 9 years had significantly shorter stays (MR = 0.79, 95% CI 0.68; 0.91, *p* = 0.001 and MR = 0.82, 95% CI 0.70; 0.96, *p* = 0.012, respectively). No significant association was observed between gender and LOS (male vs. female: MR = 1.03, 95% CI 0.97; 1.10, *p* = 0.328).

Associations with socioeconomic factors were also observed. Compared with White children, Black ethnicity was associated with a significantly longer mean LOS (MR = 1.19, 95% CI 1.10; 1.28, *p* < 0.001), whereas estimates for Romani and Asian ethnicity were not statistically significant. The main language spoken in the household was not significantly associated with LOS.

Regarding maternal education, lower educational attainment was associated with longer LOS. Compared with higher education, maternal education at the second basic cycle or lower was associated with a 47% increase in mean LOS (MR = 1.47, 95% CI 1.26; 1.72, *p* = 0.008), while secondary education was associated with an 11% increase (MR = 1.11, 95% CI 1.02; 1.21, *p* = 0.022). Maternal third basic cycle education showed borderline statistical significance (MR = 1.11, 95% CI 1.00; 1.22, *p* = 0.055), suggesting a possible gradient of increasing LOS with lower maternal education level.

In contrast, paternal education level was inversely associated with LOS. Compared with higher education, paternal education at the second basic cycle or lower was associated with shorter LOS (MR = 0.73, 95% CI 0.63; 0.84, *p* < 0.001), as was third basic cycle education (MR = 0.86, 95% CI 0.76; 0.97, *p* = 0.015). Most parental occupational categories were not associated with LOS; however, children whose fathers had skilled manual occupations had significantly longer mean stays (MR = 1.17, 95% CI 1.07; 1.28, *p* < 0.001).

A temporal decline in LOS was observed over the study period. Each one-year increase in year of admission was associated with a 3% reduction in mean LOS (MR = 0.97, 95% CI 0.96; 0.98, *p* < 0.001).

### 3.2. Admission to Intensive Care Unit

Admissions to ICU, including both pediatric and neonatal units, accounted for 7.8% (n = 628) of all hospital admissions.

In the GEE model for admission to ICU (Table 3), age also showed the strongest association. Compared with adolescents aged ≥15 years, neonatal admissions had more than 9-fold higher odds of ICU admission (OR = 9.25, 95% CI 4.84; 17.68, *p* < 0.001). No statistically significant differences were observed between the remaining pediatric age groups and the reference category.

Male gender was associated with 25% higher odds of ICU admission (OR = 1.25, 95% CI 1.01; 1.55, *p* = 0.048) compared to female gender.

Socioeconomic factors were also associated with ICU admission. Maternal education showed an inverse gradient, whereby lower educational attainment was associated with reduced odds of ICU admission. Compared with higher education, maternal education at the second basic cycle or lower was associated with a 55% reduction in the odds of ICU admission (OR = 0.45, 95% CI 0.28; 0.71, *p* < 0.001), while third basic cycle and secondary education were associated with 33% (OR = 0.67, 95% CI 0.47; 0.98, *p* = 0.037) and 31% (OR = 0.69, 95% CI 0.49; 0.98, *p* = 0.040), respectively.

In contrast, maternal unskilled occupation was associated with increased ICU admission (OR = 1.66, 95% CI 1.09; 2.53; *p* = 0.019). Paternal skilled manual occupation was likewise associated with 51% higher odds of ICU admission (OR = 1.51, 95% CI 1.07; 2.12, *p* = 0.019).

Ethnicity and language were not associated with ICU admission, although the “Other” language category showed borderline evidence of lower odds of ICU admission (OR = 0.33, 95% CI 0.11; 1.01; *p* = 0.052).

ICU admission decreased over the study period. Each one-year increase in year of admission was associated with 14% lower odds of ICU admission (OR = 0.86, 95% CI 0.80; 0.93, *p* < 0.001).

### 3.3. Health Equity Analysis: Concentration Curves and Indices

**Figure 2:**
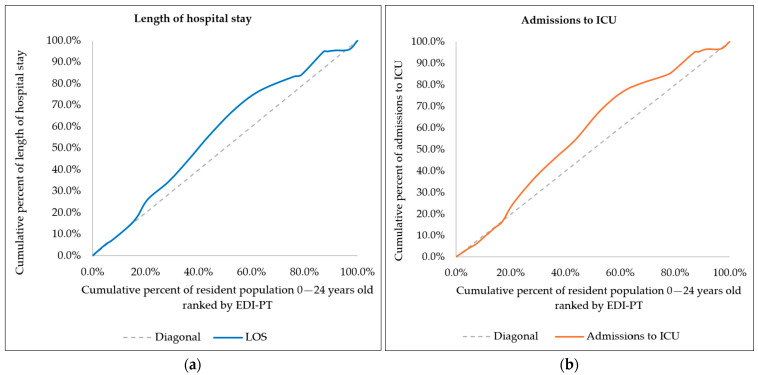
Concentration curves of (**a**) length of hospital stay (LOS) and (**b**) admission to intensive care unit (ICU) for the resident population aged 0 to 24 years ranked by European Deprivation Index—Portuguese version (EDI-PT).

Figure 2 shows the concentration curves for pediatric hospital care indicators, ranked by EDI-PT. The corresponding distributions of LOS and ICU admissions by civil parish are presented in Tables S3 and S4 in Supplementary Materials.

Both LOS and ICU admissions were disproportionately concentrated among children from more deprived civil parishes, as indicated by the concentration curves lying above the line of equality. This pattern is quantitatively reflected in the concentration indices, with values of −0.137 for LOS and −0.147 for ICU admissions, confirming a higher burden of needed hospital care in areas of greater deprivation.

However, the distribution of inequality was not uniformly monotonic across civil parishes. At the extremes of the deprivation spectrum, the curves were close to the diagonal, indicating no systematic association between deprivation and hospital care in these areas. For LOS and admission to ICU, the four most deprived and the two least deprived civil parishes showed no consistent deviation from proportionality, with the concentration curves approaching proportionality between admissions and population at both ends of the distribution.

### 3.4. Geographical Distribution of Deprivation and Hospital Care

**Figure 3:**
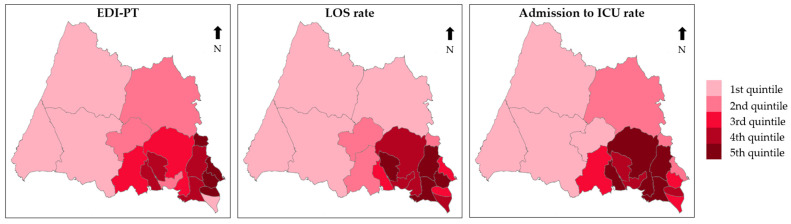
Geographical distribution, by civil parish, of European Deprivation Index—Portuguese version (EDI-PT), length of hospital stay (LOS) rate and admission to intensive care unit (ICU) rate in the civil parish population. Note: the first quintile corresponds to least deprivation.

Figure 3 presents the quintile distribution of EDI-PT scores, population-based LOS rates, and ICU admission rates by civil parish. There was a clear tendency for spatial overlap, with the eastern and most deprived civil parishes exhibiting the highest LOS and ICU admission rates, indicating that areas of greater socioeconomic deprivation experienced a higher burden of pediatric hospital care.

## 4. Discussion

This retrospective cross-sectional study examined the association between socioeconomic factors and needed hospital care—measured as LOS and admission to ICU—among pediatric patients residing in two suburban Portuguese municipalities—Amadora and Sintra—over an eight-year period. This population is particularly vulnerable, characterized by clusters of low socioeconomic status, ethnic diversity, and limited access to healthcare. Our findings confirmed the hypothesis that health inequities disproportionately affect the most deprived groups among hospitalized children in these municipalities, consistent with previous studies reporting higher hospitalization risk among children with lower socioeconomic status [6,38].

The study demonstrated concentration of needed hospital care among deprived civil parishes, as reflected by the concentration curves and negative concentration indices (−0.137 for LOS and −0.147 for ICU admissions). Notably, inequities were detected among civil parishes with intermediate deprivation, which encompass the largest portion of the population. The use of the EDI-PT, a validated instrument correlating with both objective and subjective measures of poverty [36], strengthens the reliability and comparability of these results.

These findings were consistent with the geographical small-area analysis, showing spatial overlap between deprivation and higher LOS and ICU admission rates, particularly in the eastern and most deprived civil parishes, which are predominantly urban and densely populated. This aligns with studies in other settings, such as Andrist et al. (2019) [39], who found correspondence between child poverty and ICU admissions in Cincinnati, USA, and a European systematic review reporting that neighborhood deprivation and urban density are associated with adverse child health outcomes [38].

However, complete geographical correspondence between deprivation and hospital care was not observed for all civil parishes. The concentration curves for both LOS and ICU admissions approached proportionality at the extremes, suggesting no systematic association in the four most deprived and the two least deprived parishes. This likely reflects heterogeneity within civil parishes, where disadvantaged and privileged neighborhoods coexist. Furthermore, the asymmetrical distribution of deprivation, with a substantial proportion of the population concentrated in the most disadvantaged quintile, reduced the ability to detect clear and robust socioeconomic gradients across deprivation categories. Despite this limitation, the study highlights priority areas for public health interventions.

The year of admission was included in the multivariate models to account for temporal changes in hospital care over the eight-year study period. Both LOS and the odds of admission to ICU decreased over time, consistent with broader trends in hospital care that may reflect changes in clinical practice, discharge planning, and/or case mix throughout the study period.

Among demographic and socioeconomic factors, neonatal age, parental education and paternal occupation were significantly associated with both LOS and ICU admission. Additionally, Black ethnicity was associated with higher mean LOS ratio, while male gender and maternal unskilled occupation had higher ICU admission odds. These results are consistent with extensive literature demonstrating that lower socioeconomic status is associated with worse child health and higher healthcare utilization [6,7,38,40,41,42]. Maternal education, paternal occupation, and family income have been pointed out as key determinants of child health inequities [18].

Neonatal age was associated with the highest mean LOS ratio and the highest odds of admission to ICU, likely reflecting the prolonged and severe hospitalizations associated with neonatal conditions, including prematurity, low birth weight, congenital malformations, and infections [9,43].

Only Black ethnicity was significantly associated with LOS, with a 19% higher mean ratio compared to White ethnicity (MR = 1.19; 95% CI 1.10; 1.28, *p* < 0.001). This finding may reflect the high proportion of immigrants and individuals of African descent in the two municipalities included in the study, together with the socioeconomic and biological factors associated with these populations. In particular, sickle cell disease, which is more prevalent among children of African descent and often requires prolonged hospitalization, may have contributed to the observed association between Black ethnicity and longer LOS [44].

Given that the study population was predominantly composed of White and Black ethnic groups (96.8%), the statistical power to detect associations involving other ethnicities was inherently limited.

Interestingly, ethnicity was not significantly associated with ICU admission, which contrasts with previous studies reporting ethnic disparities in ICU utilization [45]. This divergence may partly reflect the small proportion of ICU admissions (7.8%) and the predominance of White and Black patients in this group (98.4%). Additionally, factors contributing to prolonged hospital stay may differ from those associated with ICU admission. For example, chronic conditions requiring extended management, discharge barriers, and differential access to timely outpatient care may influence LOS without necessarily increasing the likelihood of ICU admission.

Several mechanisms have been proposed to explain ethnic health inequities. Immigrants and ethnic minority groups are at higher risk of economic deprivation and social exclusion, often facing discrimination, lower income, poorer housing conditions, and reduced access to education and healthcare [46,47,48]. Some studies have shown that these disparities persist even after adjustment for socioeconomic status [49,50], suggesting an independent effect of ethnic discrimination. In the municipalities of Amadora and Sintra, limited access to a family doctor disproportionately affects immigrant populations [16,46]. This may contribute to delayed access to care, higher out-of-pocket health expenditures and greater severity of illness at hospital presentation, further exacerbating socioeconomic and health disparities [51].

Similar mechanisms may also underlie the effects of language barriers on healthcare access. Limited proficiency in the dominant language of a country has been linked to lower access to healthcare, poorer physical and developmental health, increased hospitalization and ICU admissions and longer LOS among children [52,53]. However, our study did not show an association between language and hospital care variables. This could be due to the fact that families from Portuguese-speaking African countries, who represented a substantial proportion of the study population, are fluent in Portuguese as a second language.

There is substantial evidence showing that lower parental education, particularly maternal education, is associated with worse health outcomes in children [18,54]. In our study, maternal education showed an inconsistent association with hospital care needs. Lower maternal education was associated with longer LOS, with maternal education at the second basic cycle or lower associated with a 47% increase in mean LOS, while secondary education was associated with an 11% increase. Additionally, a gradient pattern was observed, whereby lower maternal educational attainment tended to be associated with progressively longer LOS, with third basic cycle education showing borderline statistical significance. These findings reinforce the central role of mothers in childcare and child health equity.

Unexpectedly, lower maternal education was associated with lower odds of ICU admission. This apparent inconsistency should be interpreted with caution. The relatively low prevalence of ICU admission in our sample may have limited the ability to identify a consistent association. Moreover, the findings may reflect information bias related to the high proportion of mother’s education level missing data (18.7%) or residual confounding by clinical factors not captured in administrative data.

Regarding fathers’ education, only secondary level showed a significant association with LOS, corresponding to a 14% lower mean LOS compared to higher educational attainment. The relatively low proportion of fathers with higher education level (12.1%) might have limited the statistical power of the model to detect additional associations involving this variable. Nevertheless, there is a wide body of evidence consistently reporting a stronger contribution of maternal education to child health outcomes compared with paternal education [18,54].

Our findings also demonstrated significant associations between parental occupational status and hospital care indicators. Maternal unskilled occupation was associated with a 1.66-fold increase in the odds of ICU admission. In addition, paternal skilled manual occupation was associated with a 17% higher mean LOS and 51% higher odds of admission to ICU. Parental manual and unskilled jobs are often linked to reduced availability for childcare, lower health literacy, and financial constraints, perpetuating cycles of disadvantage from childhood into adulthood [55,56,57,58].

### 4.1. Limitations

This study has some limitations. First, it is retrospective and relies on a pre-existing hospital database that lacks key variables such as family income, immigration status, and access to primary healthcare, limiting the ability to fully capture socioeconomic determinants. Additionally, the models might have been improved by including adjustment for the clinical severity of children at hospital presentation. However, it should be noted that the dependent variables themselves—LOS and ICU admission—partially reflect clinical severity. Moreover, the administrative database did not include appropriate indicators of baseline clinical severity that could be incorporated into the analysis.

Second, a considerable proportion of missing data was observed for some socioeconomic variables, particularly parental education. This was addressed through multiple imputation, a method recommended in epidemiological research because it preserves statistical power and may reduce bias compared with complete case analysis when the missingness can be explained by observed data. In our imputation models, we included the outcomes and all covariates used in the analyses, which is consistent with guidance to make the MAR assumption more plausible by conditioning on predictors of missingness and outcomes.

Nevertheless, as in most observational studies, missing not at random (MNAR) cannot be fully excluded, particularly for sensitive socioeconomic indicators. If nonresponse occurred more frequently among families with lower education, or in situations involving greater clinical severity where data collection may have been less complete, residual information bias could remain. In practical terms, such mechanisms would most often be expected to dilute socioeconomic gradients, suggesting that the observed inequalities may represent conservative estimates; however, the exact direction and magnitude of any MNAR-related bias cannot be determined from the available data alone.

Future studies should prioritize strategies to minimize missingness in socioeconomic variables (e.g., culturally adapted data collection procedures and language support), and prospective designs with specifically tailored databases, incorporating a broader range of socioeconomic indicators.

Third, the study did not distinguish between medical and social admissions, which may represent potential outliers. Although pediatric admissions exclusively for social reasons are a minority (0.3–1.2%) [59,60], they can be prolonged and influence overall LOS estimates.

Fourth, the relatively low frequency of ICU admissions in the sample (7.8%) may have reduced the statistical power of the multivariate model, potentially limiting the ability to detect associations between socioeconomic determinants and this dependent variable. Future studies including a broader range of socioeconomic covariates and larger samples of critically ill children may improve the capacity of analytical models to better explain socioeconomic differences in ICU admission.

Finally, limitations arise from the ecological data and administrative boundaries used. The 2013 reorganization of civil parishes and subsequent recalculation of EDI-PT scores may have introduced bias, as the recalculation process is not fully described [36]. Additionally, the study period (2014–2021) falls between two national censuses, while population denominators were based on the 2021 Census, potentially affecting representativeness.

Another limitation of the ecological analysis is the limited ability of the EDI-PT to capture geographical socioeconomic heterogeneity, as suggested by the near proportionality observed at the extremes of the concentration curves. Analysis at smaller geographical scale (e.g., neighborhood level), potentially incorporating alternative deprivation indices and indicators such as income, education, housing quality, access to essential services, and transportation, could provide a more comprehensive understanding of the determinants underlying localized health inequities.

Moreover, the findings from ecological analysis should be interpreted with caution at the individual level to avoid ecological fallacy. Future studies using multilevel analytical approaches may help overcome this limitation and provide a more robust understanding of individual- and area-level determinants of hospital care.

### 4.2. Implications for Public Health Policy

Reducing health inequities requires public policies spanning the entire life course, beginning at pre-conception and addressing the multiple dimensions of children’s socioeconomic circumstances, including parental background [61]. Strategies should integrate social policies and public health interventions under a coordinated Health in All Policies framework [4,62]. Proportionate universalism, combining universal services with programs targeted at the most disadvantaged, which has been widely advocated [4,63], should also be considered.

Ensuring early and universal access to childcare and education is among the most powerful measures to promote equity [64]. Supporting income, education, and labor market participation, particularly for mothers, immigrants, and other disadvantaged groups, can act synergistically to reduce inequities [65]. In parallel, universal healthcare access and established public health surveillance and prevention programs remain essential [4,62].

Community-level interventions are critical for early identification of vulnerabilities and delivery of care. Primary healthcare centers, schools, home visits, and local organizations provide effective platforms for outreach [65,66]. In regions with high cultural diversity and immigration, integration programs should be prioritized, combining social and employment support, language acquisition, school enrollment, and targeted public health initiatives [67].

Addressing deprived areas requires improving both neighborhood infrastructure and access to essential services. Health-friendly urban planning should consider housing quality, safety, green spaces, and transportation [38]. Access to healthcare, childcare, education, welfare, and employment also functions as local social determinants of health [20]. Beck et al. (2019) propose a comprehensive intervention model that effectively reduced hospitalization rates in high-risk neighborhoods [68]. This model combines clinical care interventions (preventive care for chronic conditions, multidisciplinary and transitional care) with social risk mitigation and continuity of care, linking hospitals, families, schools, and community agencies [68]. A successful reduction in geographical inequities also requires active involvement of local political councils and community organizations.

## 5. Conclusions

This study identified significant inequities in pediatric hospital care associated with socioeconomic deprivation in the Portuguese municipalities of Amadora and Sintra. Longer hospital stays and ICU admissions were disproportionately concentrated in deprived civil parishes. Black ethnicity and parents’ unskilled or manual occupations were associated with greater hospital care needs.

These findings highlight the critical role of socioeconomic factors in shaping child health, using two municipalities characterized by socioeconomic vulnerability, immigration, and ethnic diversity as illustrative case studies. The results enhance understanding of how social determinants are associated with health outcomes and how the socioeconomic gradient is linked to health inequities, even within urban areas of a high-income country.

Childhood represents a period of heightened susceptibility to socioeconomic disadvantage. Public health policies should leverage this unique window of opportunity to interrupt cycles of cumulative adversity and reduce lifelong inequities through early, targeted interventions aimed at addressing patterns of disadvantage from the earliest stages of life.

## Figures and Tables

**Table 1:** Demographic and socioeconomic characteristics of admissions (n = 8016).

Variable/Category	Frequency	%
Age group		
<28 days	877	10.9
28 days–11 months	2025	25.3
1–4 years	2212	27.6
5–9 years	1099	13.7
10–14 years	947	11.8
≥15 years	856	10.7
Gender		
Male	4326	54.0
Female	3690	46.0
Ethnicity		
White	4085	50.9
Black	3678	45.9
Romani	151	1.9
Asian	102	1.3
Language		
Portuguese	7607	94.9
Creole or Fulah	174	2.2
Asian	114	1.4
Other	121	1.5
Mother’s education level		
2nd basic or lower	1768	22.0
3rd basic	2435	30.4
Secondary	2556	31.9
Higher	1257	15.7
Father’s education level		
2nd basic or lower	1684	21.0
3rd basic	2831	35.3
Secondary	2531	31.6
Higher	970	12.1
Mother’s occupation		
White-collar	1627	20.3
Armed forces, security, personal care and sales	2252	28.1
Skilled manual	137	1.7
Unskilled	1583	19.7
Not working	2417	30.2
Father’s occupation		
White-collar	2002	25.0
Armed forces, security, personal care and sales	1492	18.6
Skilled manual	2436	30.4
Unskilled	1001	12.5
Not working	1085	13.5

**Table 2:** Multivariate model of length of hospital stay (n = 8016).

Independent Variable/Category	LOS, Days Median (IQR)	MR (Exp(β))	95% CI	*p*
Age group				
<28 days	8.74 (4.20; 14.88)	2.29	1.96; 2.67	<0.001
28 days–11 months	4.00 (2.18; 6.25)	0.95	0.82; 1.10	0.458
1–4 years	3.00 (2.00; 5.03)	0.79	0.68; 0.91	0.001
5–9 years	3.16 (2.00; 6.00)	0.82	0.70; 0.96	0.012
10–14 years	4.00 (2.00; 7.00)	0.95	0.82; 1.11	0.528
≥15 years	4.00 (2.00; 7.08)	1.00		
Gender				
Male	4.00 (2.00; 7.00)	1.03	0.97; 1.10	0.328
Female	4.00 (2.00; 6.82)	1.00		
Ethnicity				
White	3.40 (2.00; 6.00)	1.00		
Black	4.00 (2.04; 8.00)	1.19	1.10; 1.28	<0.001
Romani	4.00 (2.47; 7.00)	0.97	0.79; 1.19	0.777
Asian	4.00 (2.93; 8.00)	1.30	0.90; 1.88	0.160
Language				
Portuguese	4.00 (2.00; 7.00)	1.00		
Creole or Fulah	4.00 (2.56; 9.00)	1.22	0.84; 1.78	0.299
Asian	5.00 (2.72; 9.65)	0.95	0.73; 1.24	0.702
Other	4.00 (2.04; 8.00)	1.12	0.85; 1.48	0.426
Mother’s education level				
2nd basic or lower	4.06 (2.10; 8.00)	1.47	1.26; 1.72	0.008
3rd basic	4.00 (2.00; 7.00)	1.11	1.00; 1.22	0.055
Secondary	3.83 (2.00; 6.84)	1.11	1.02; 1.21	0.022
Higher	3.88 (2.00; 6.96)	1.00		
Father’s education level				
2nd basic or lower	4.00 (2.00; 7.00)	0.73	0.63; 0.84	<0.001
3rd basic	3.90 (2.00; 7.00)	0.86	0.76; 0.97	0.015
Secondary	4.00 (2.00; 7.00)	0.92	0.82; 1.03	0.158
Higher	4.00 (2.00; 7.00)	1.00		
Mother’s occupation				
White-collar	3.78 (2.00; 6.00)	1.00		
Armed forces, security, personal care and sales	3.80 (2.00; 6.83)	0.96	0.88; 1.04	0.331
Skilled manual	3.84 (2.00; 7.00)	0.99	0.82; 1.19	0.926
Unskilled	4.00 (2.00; 8.00)	1.10	0.95; 1.26	0.198
Not working	4.00 (2.06; 7.14)	1.05	0.96; 1.16	0.294
Father’s occupation				
White-collar	3.92 (2.00; 6.13)	1.00		
Armed forces, security, personal care and sales	3.87 (2.00; 7.00)	1.06	0.97; 1.16	0.206
Skilled manual	4.00 (2.00; 7.01)	1.17	1.07; 1.28	<0.001
Unskilled	4.00 (2.00; 7.15)	1.04	0.93; 1.17	0.455
Not working	4.00 (2.04; 7.17)	1.07	0.95; 1.22	0.274
Year of admission		0.97	0.96; 0.98	<0.001

CI, confidence interval; Exp(β), exponentiated coefficient; IQR, interquartile range; MR, mean ratio. MR = 1 corresponds to the reference category.

**Table 3:** Multivariate model of admission to intensive care unit (n = 8016).

Independent Variable/Category	Frequency (% Within Category of Independent Variable)	OR (Exp(β))	95% CI	*p*
Age group				
<28 days	315 (35.9%)	9.25	4.84; 17.68	<0.001
28 days–11 months	78 (3.9%)	0.55	0.28; 1.10	0.092
1–4 years	83 (3.8%)	0.62	0.31; 1.24	0.178
5–9 years	50 (4.5%)	0.75	0.33; 1.70	0.494
10–14 years	53 (5.6%)	0.42	0.10; 1.81	0.248
≥15 years	49 (5.7%)	1.00		
Gender				
Male	376 (8.7%)	1.25	1.01; 1.55	0.048
Female	252 (6.8%)	1.00		
Ethnicity				
White	284 (7.0%)	1.00		
Black	334 (9.1%)	1.09	0.86; 1.38	0.472
Romani	3 (2.0%)	0.34	0.09; 1.28	0.113
Asian	7 (6.9%)	1.16	0.42; 3.23	0.773
Language				
Portuguese	580 (7.6%)	1.00		
Creole or Fulah	16 (9.2%)	1.07	0.56; 2.04	0.831
Asian	28 (24.6%)	1.22	0.44; 3.34	0.705
Other	4 (3.3%)	0.33	0.11; 1.01	0.052
Mother’s education level				
2nd basic or lower	111 (6.3%)	0.45	0.28; 0.71	<0.001
3rd basic	195 (8.0%)	0.67	0.47; 0.98	0.037
Secondary	183 (7.2%)	0.69	0.49; 0.98	0.040
Higher	139 (11.1%)	1.00		
Father’s education level				
2nd basic or lower	105 (6.2%)	0.93	0.55; 1.56	0.786
3rd basic	224 (7.9%)	0.09	0.71; 1.67	0.703
Secondary	213 (8.4%)	0.98	0.65; 1.49	0.940
Higher	86 (8.9%)	1.00		0.657
Mother’s occupation				
White-collar	137 (8.4%)	1.00		
Armed forces, security, personal care and sales	151 (6.7%)	1.14	0.80; 1.61	0.479
Skilled manual	10 (7.3%)	1.43	0.65; 3.13	0.369
Unskilled	145 (9.2%)	1.66	1.09; 2.53	0.019
Not working	185 (7.6%)	1.30	0.90; 1.89	0.168
Father’s occupation				
White-collar	137 (6.8%)	1.00		
Armed forces, security, personal care and sales	137 (9.2%)	1.35	1.95; 1.91	0.091
Skilled manual	212 (8.7%)	1.51	1.07; 2.12	0.019
Unskilled	73 (7.3%)	1.17	0.76; 1.81	0.475
Not working	69 (6.4%)	1.30	0.86; 1.95	0.211
Year of admission		0.86	0.80; 0.93	<0.001

CI, confidence interval; Exp(β), exponentiated coefficient; ICU, intensive care unit; OR, odds ratio. OR = 1 corresponds to the reference category.

## Data Availability

The datasets generated and analyzed during the current study are not publicly available because they contain participants’ sensitive data, which the authors do not have permission to share.

## Supplementary Materials

The following supporting information can be downloaded at: https://www.mdpi.com/article/10.3390/ijerph23060767/s1, Table S1: Number and proportion of valid and missing cases per variable (n = 8016), Table S2: Number of residents in 2021 and European Deprivation Index in 2013 by civil parish of the municipalities of Amadora and Sintra, Table S3: Concentration index and distribution of length of hospital stay by civil parish, Table S4: Concentration index and distribution of admissions to intensive care unit by civil parish. References [13,37] are cited in the Supplementary Materials.

## Abbreviations

The following abbreviations are used in this manuscript:

EU	European Union
LOS	Length of hospital stay
ICU	Intensive care unit(s)
MAR	Missing at random
PMM	Predictive mean matching
GEE	Generalized estimating equations
Exp(β)	Exponentiated coefficient
MR	Mean ratio
OR	Odds ratio
CI	Confidence interval(s)
EDI-PT	European Deprivation Index—Portuguese Version
IQR	Interquartile range
MNAR	Missing not at random
