# Supplementary material for: The Impact of Socioeconomic Inequities and Small-Area Deprivation on Child Inpatient Care: Evidence from a Quantitative Study in a Vulnerable Suburban Setting
Source: Int J Environ Res Public Health. 2026 Jun 7;23(6):767. doi: 10.3390/ijerph23060767 (PMC13300299; doi:10.3390/ijerph23060767)
Supplement: Supplementary file 1 [file ijerph-23-00767-s001.zip › ijerph-4252681-supplementary.pdf]

Table S1: Number and proportion of valid and missing cases per variable (n = 8016).

Variable	Valid	Missing	Percentage of missing cases
Father's education level	5880	2136	26.6%
Mother's education level	6515	1501	18.7%
Father's occupation	6947	1069	13.3%
Ethnicity	7060	956	11.9%
Father's employment status	7347	669	8.3%
Mother's occupation	7512	504	6.3%
Language spoken at the household	7651	365	4.6%
Mother's employment status	7695	321	4.0%

Table S2: Number of residents in 2021 and European Deprivation Index in 2013 by civil parish of the municipalities of Amadora and Sintra.

Municipality	Civil parish	**Total residents** [13]	**Residents 0—24 yo** [13]	**EDI-PT** [37]
Amadora	Alfragide	16,837	4662	–0.389
	Mina de Água	42,961	11,243	5.224
	Venteira	26,168	5719	5.849
	Falagueira—Venda Nova	20,788	4670	7.055
	Águas Livres	37,607	8979	7.314
	Encosta do Sol	27,093	7136	8.887
	Total of Amadora	171,454	42,409	
Sintra	Sintra	29,896	7691	–0.055
	São João das Lampas e Terrugem	17,993	4487	2.118
	Colares	7,746	1820	2.437
	Massamá e Monte Abraão	47,804	12,293	2.739
	Almargem do Bispo, Pêro Pinheiro e Montelavar	17,262	4246	3.196
	Algueirão—Mem Martins	68,649	18,869	3.251
	Rio de Mouro	49,489	13,939	3.451
	Queluz e Belas	52,414	14,053	4.066
	Agualva e Mira Sintra	41,323	10,673	5.098
	Cacém e São Marcos	39,683	11,495	5.225
	Casal de Cambra	13,347	3,960	7.957
	Total of Sintra	385,606	103,526	
Total of Amadora and Sintra		557,060	145,935	

EDI-PT, European Deprivation Index—Portuguese version; yo, years old.

EDI-PT has the following quintile distribution (from least to most deprived): 1 (–8.155 to –1.774); 2 (–1.773 to –0.605); 3 (–0.604 to 0.338); 4 (0.339 to 1.581); and 5 (1.582 to 17.249).2. Fifteen of the 17 civil parishes in Amadora and Sintra are fifth quintile EDI-PT (most deprived), corresponding to 91.5% of the population aged 0 to 24 years; the other two civil parishes are third quintile EDI-PT score.

Table S3: Concentration index and distribution of length of hospital stay by civil parish.

Civil parish ranked by EDI-PT (from most to least deprived)	% of residents 0—24 years old	Number of days of LOS	LOS rate among residents *	% of days of LOS **	Concentration index
Encosta do Sol	4.9%	2687	0.38	5.18%	
Casal de Cambra	2.7%	1149	0.29	2.22%	
Águas Livres	6.2%	3307	0.37	6.38%	
Falag.—VN	3.2%	2325	0.50	4.48%	
Venteira	3.9%	4131	0.72	7.96%	
CSM	7.9%	4275	0.37	8.24%	
Mina de Água	7.7%	5407	0.48	10.42%	
AMS	7.3%	5498	0.52	10.60%	
QB	9.6%	6370	0.45	12.28%	
RDM	9.6%	4570	0.33	8.81%	
AMM	12.9%	3436	0.18	6.63%	
APM	2.9%	436	0.10	0.84%	
MMA	8.4%	5612	0.46	10.82%	
Colares	1.2%	34	0.02	0.07%	
SJLT	3.1%	246	0.05	0.47%	
Sintra	5.3%	212	0.03	0.41%	
Alfragide	3.2%	2169	0.47	4.18%	
Total	100.0%	51,864		100.0%	–0.137

AMM, Algueirão—Mem Martins; AMS, Agualva e Mira Sintra; APM, Almargem do Bispo, Pêro Pinheiro e Montelavar; CSM, Cacém e São Marcos; EDI-PT, European Deprivation Index—Portuguese version; Falag.-VN, Falagueira—Venda Nova; LOS, length of hospital stay; MMA, Massamá e Monte Abraão; QB, Queluz e Belas; RDM, Rio de Mouro; SJLT, São João das Lampas e Terrugem.

* (Number of days of LOS) / (Number of residents 0-24 years old in the civil parish)

** (Number of days of LOS) / (Total days of LOS)

Table S4: Concentration index and distribution of admissions to intensive care unit by civil parish.

Civil parish ranked by EDI-PT (from most to least deprived)	% of residents 0—24 years old	Number of admissions to ICU	% admissions to ICU among residents *	% admissions to ICU **	Concentration index
Encosta do Sol	4.9%	26	0.36%	4.14%	
Casal de Cambra	2.7%	13	0.33%	2.07%	
Águas Livres	6.2%	44	0.49%	7.01%	
Falag. - VN	3.2%	21	0.45%	3.34%	
Venteira	3.9%	52	0.91%	8.28%	
CSM	7.9%	75	0.65%	11.94%	
Mina de Água	7.7%	59	0.52%	9.39%	
AMS	7.3%	53	0.50%	8.44%	
QB	9.6%	89	0.63%	14.17%	
RDM	9.6%	59	0.42%	9.39%	
AMM	12.9%	37	0.20%	5.89%	
APM	2.9%	11	0.26%	1.75%	
MMA	8.4%	59	0.48%	9.39%	
Colares	1.2%	0	0.00%	0.00%	
SJLT	3.1%	8	0.18%	1.27%	
Sintra	5.3%	1	0.01%	0.16%	
Alfragide	3.2%	21	0.45%	3.34%	
Total	100.0%	628		100.0%	–0.147

AMM, Algueirão—Mem Martins; AMS, Agualva e Mira Sintra; APM, Almargem do Bispo, Pêro Pinheiro e Montelavar; CSM, Cacém e São Marcos; EDI-PT, European Deprivation Index—Portuguese version; Falag.-VN, Falagueira—Venda Nova; ICU, intensive care unit; MMA, Massamá e Monte Abraão; QB, Queluz e Belas; RDM, Rio de Mouro; SJLT, São João das Lampas e Terrugem.

* (Number of admissions to ICU) / (Number of residents 0–24 years old in the civil parish)

** (Number of admissions to ICU) / (Total admissions to ICU)
